# Prenatal Screening of Trisomy 21: Could Oxidative Stress Markers Play a Role?

**DOI:** 10.3390/jcm10112382

**Published:** 2021-05-28

**Authors:** Angelika Buczyńska, Iwona Sidorkiewicz, Sławomir Ławicki, Adam Jacek Krętowski, Monika Zbucka-Krętowska

**Affiliations:** 1 Clinical Research Centre, Medical University of Bialystok, 15-276 Bialystok, Poland; angelika.buczynska@umb.edu.pl (A.B.); iwona.sidorkiewicz@umb.edu.pl (I.S.); adamkretowski@wp.pl (A.J.K.); 2 Department of Population Medicine and Civilization Diseases Prevention, Medical University of Bialystok, 15-276 Bialystok, Poland; slawicki@umb.edu.pl; 3 Department of Endocrinology, Diabetology and Internal Medicine, Medical University of Bialystok, 15-276 Bialystok, Poland; 4 Department of Gynecological Endocrinology and Adolescent Gynecology, Medical University of Bialystok, 15-276 Bialystok, Poland

**Keywords:** trisomy 21, Down syndrome, oxidative stress, antioxidant protein, prenatal screening

## Abstract

Despite significant progress in trisomy 21 (T21) diagnostic tools, amniocentesis is still used for the confirmation of an abnormal fetal karyotype. Invasive tests carry the potential risk of miscarriage; thus, screening biomarkers are commonly used before undergoing invasive procedures. In our study, we investigated the possible application of oxidative stress markers in the prenatal screening of trisomy 21. The DNA/RNA oxidative stress damage products (OSDPs), advanced glycation end (AGE) products, ischemia-modified albumin (IMA), alfa-1-antitrypsin (A1AT), asprosin, and vitamin D concentrations were measured in both maternal plasma and amniotic fluid in trisomy 21 (T21) and euploid pregnancies. The obtained results indicated increased levels of DNA/RNA OSDPs and asprosin with simultaneous decreased levels of vitamin D and A1AT in the study group. The diagnostic utility of the plasma measurement based on the area under the received operative characteristic (ROC) curve (AUC) calculation of asprosin (AUC = 0.965), IMA (AUC = 0.880), AGE (AUC = 0.846) and DNA/RNA OSDPs (AUC = 0.506) in T21 screening was demonstrated. The obtained results indicate a potential role for the application of oxidative stress markers in the prenatal screening of T21 with the highest screening utility of plasma asprosin.

## 1. Introduction

Trisomy 21 (T21), also known as Down syndrome, is an autosomal aneuploidy, appearing in 1/700 live births. An additional copy of chromosome 21 is a result of the incorrect separation during gametogenesis (95% of patients) [1,2,3]. Trisomy 21 is a complex condition associated with congenital anomalies, which include intellectual developmental disorder, congenital heart defects, gastrointestinal anomalies, immune system defects, thyroid disease, bone defects, genitourinary system defects, strabismus, and many other diseases. Additionally, an increased risk of many chronic diseases typically associated with older age such as Alzheimer’s disease, dementia, and obesity is observed [4]. In T21 prenatal screening, serum biomarkers combined with ultrasound examination and cell-free fetal DNA are used to calculate the risk of T21 occurrence [5,6,7,8,9]. Despite the fact that cell-free fetal DNA evaluation is characterized by high accuracy (almost 99%), it is still combined with high costs which has not yet allowed for very wide diffusion to the general population, or acceptance by various national healthcare systems into their protocols [10]. Furthermore, mothers with a high calculated risk of trisomy 21 (either by combined test and/or cell-free fetal DNA) should be counselled, and invasive testing, such as chorionic villus sampling (CVS) and/or amniocentesis, should be offered. The application of novel biochemical screening markers may result in the elevation of the sensitivity and specificity of noninvasive prenatal tests and may reduce the unjustified use of invasive procedures while simultaneously decreasing the risk of miscarriage, combined with the use of invasive tests [11]. Data in the literature underline the connection between fetal chromosomal aberrations and disturbances in oxidative stress with antioxidant processes [12,13,14,15]. It was previously hypothesized that the upregulated oxidative stress level is related to T21 pathogenesis; this was later proved by Žitňanová et al., who demonstrated upregulated levels of oxidative stress markers measured in T21 individuals [16]. Thus, it seems necessary to evaluate the hypothesis about oxidative stress biomarkers in T21 prenatal screening. Considering the fact that crucial genes of the oxidative stress pathway are mapped on chromosome 21 [17], the hypothesis of the significance of oxidative stress, not only in T21 postnatal pathology but also in prenatal diagnosis, needs to be evaluated. Accordingly, the biomarkers of oxidative stress measurements could be relevant in the screening of T21 [18]. The aim of this study was to assess the utility of selected parameters of oxidative stress markers in maternal plasma and amniotic fluid for T21 screening. DNA/RNA oxidative stress damage products (OSDPs), as well as other commonly used oxidative stress markers (ischemia-modified albumin (IMA) and advanced glycation ends products (AGE)), were evaluated in this study. Furthermore, novel antioxidant proteins—asprosin and alfa-1-antitrypsin (A1AT)—and vitamin D were also assessed and compared between T21 and euploid pregnancies.

## 2. Materials and Methods

### 2.1. Experimental Overview—Patient Recruitment

This was a prospective case–control study. The study and control groups consisted of women who underwent routine amniocentesis between the 15th and 18th weeks of gestation at the Department of Reproduction and Gynecological Endocrinology of the Medical University of Bialystok, Poland. A total amount of 100 pregnant women underwent screening procedures between 2017 and 2020, and 40 were included and recruited for the subsequent evaluation. The increased risk of chromosomal aberrations in noninvasive prenatal screening and an age greater than 35 years were indications for amniocentesis. Chronic or acute diseases, hormonal treatment, anti-inflammatory treatment, high-risk pregnancy, and preterm delivery in the patient’s medical history were the exclusion criteria [19]. All participants were aware of the potential risks prior to the amniocentesis procedure and received relevant and necessary information about the study. The study group did not differ with respect to the course of pregnancy and body mass index (BMI). A necessary sample size to detect the significant differences in all studied parameters between groups was confirmed using power analysis [20]. Considering a 5% margin of error and 95% confidence level, the recommended sample size of our preliminary study was 16. Following karyotype test analysis, 20 women carrying T21 fetuses and 20 women with euploid fetuses qualified for the study. All participants had 5.5 ml of venous blood drawn on the day of amniocentesis. The biological material was centrifuged, with subsequent plasma separation, and frozen at −80 °C. Amniotic fluid samples with possible blood contamination were excluded from the study.

### 2.2. Ethics Statement

The experimental protocol was approved by the Bioethics Committee of the Medical University of Bialystok, Poland (APK/002/351/2020), and confirmation consent was received from each participant.

### 2.3. Laboratory Examinations

The IMA, AGE, A1AT, and asprosin concentrations were measured using an enzyme-linked immunosorbent assay (ELISA) (Enzyme-linked Immunosorbent Assay Kit; Cloud-Clone Corp., Wuhan, China; CEA825Hu, CEB353Ge, SEB697Hu, and SEA332Hu, respectively) according to the manufacturer’s instructions. The DNA/RNA OSDP concentrations were assayed using an immunoassay kit (DNA/RNA Oxidative Damage (High Sensitivity) ELISA Kit, Cayman Chemicals, Ann Arbor, Michigan, MI, USA, 589320). This kit enabled the simultaneous detection of DNA/RNA OSDPs, such as 8-hydroxyguanosine (8-OHG), 8-hydroxy-2’-deoxyguanosine (8-OHdG), and 8-hydroxyguanine. The vitamin D concentration was evaluated using a commercial kit for 25-OH Vitamin D Total ELISA (Gentaur, Sopot, Poland, KAP1971). The total vitamin D measurement was evaluated through the chemiluminescence method using Cobas E411, from Roche company (07464215). The samples and controls were randomized, then measured in the same run, using the blind analysis method.

### 2.4. Data Management and Statistical Analysis

Statistical analyses were performed using Statistica 13.3 (StatSoft, Tibco Software Inc., Palo Alto, CA, USA) and GraphPad Prism v. 9.0 (GraphPad Software, Inc., San Diego, CA, USA). During the analysis, the lack of data distribution normality was demonstrated using the Shapiro–Wilk test. Thus, the groups were compared using the nonparametric Mann–Whitney test, and *p* < 0.05 was considered statistically significant. The Spearman test for multiple comparisons was used to perform correlation analyses between the concentrations of all the studied parameters in plasma and amniotic fluid samples. In addition, the receiver operating characteristic (ROC) curves were determined with simultaneous sensitivity and specificity calculations. Screening cutoff points were determined using Youden’s index [21]. Odds ratios (ORs) were calculated using commercially available MedCalc software [22].

## 3. Results

### 3.1. The Comparison of Oxidative Stress-Related Parameters between the Study and Control Groups

Following the oxidative stress marker analyses, the concentrations of the DNA/RNA OSDPs were found to be significantly higher in amniotic fluid samples in T21 individuals compared to those from the control group (*p* < 0.05). No significant difference was observed in the plasma concentrations of the DNA/RNA OSDPs between the study and control groups. In the T21 group, the AGE concentrations were found to be significantly lower in both plasma and amniotic fluid samples compared to those from healthy subjects (*p* < 0.001). Additionally, the maternal plasma IMA concentrations were also lower in the T21 group in comparison to control group (*p* < 0.0001).

Considering the antioxidant parameters assessed in the study group, the total vitamin D plasma concentrations were significantly lower when compared to the control group (*p* < 0.05). To verify vitamin D deficiency, the 25-OH vitamin D concentrations were measured in plasma and amniotic fluid samples. Significant differences between the study and control groups were not proven, but 25-OH vitamin D levels lower than recommended were observed in both groups. 

**Figure 1:**
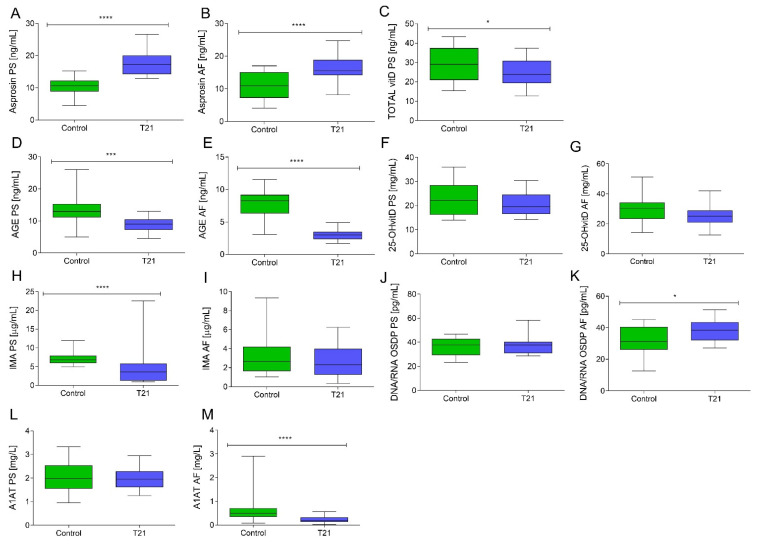
The studied protein concentrations measured in the plasma and amniotic fluid samples. Different asterisks above the bars indicate significant differences compared to the control (* *p* ≤ 0.05; *** *p* ≤ 0.001; **** *p* ≤ 0.0001). (**A**) Plasma asprosin; (**B**) amniotic fluid asprosin; (**C**) plasma total vitamin D; (**D**) plasma advanced glycation end products; (**E**) amniotic fluid advanced glycation end products; (**F**) plasma 25-OH vitamin D; (**G**) amniotic fluid 25-OH vitamin D; (**H**) plasma ischemia-modified albumin; (**I**) amniotic fluid ischemia-modified albumin; (**J**) plasma DNA/RNA oxidative stress damage product; (**K**) amniotic fluid DNA/RNA oxidative stress damage products; (**L**) plasma alfa-1-antitrypsin; (**M**) amniotic fluid alfa-1-antitrypsin. A1AT, alfa-1-antitrypsin; AF, amniotic fluid; AGE, advanced glycation end products; Control, control group; IMA, ischemia-modified albumin; OSDP, oxidative stress damage product; PS, plasma; T21, trisomy 21.

Novel antioxidant protein concentrations were also determined. The study group asprosin concentrations were significantly higher in both plasma and amniotic fluid samples compared to euploid pregnancies (*p* < 0.001). Interestingly, the A1AT concentrations were found to be significantly lower in amniotic fluid samples in the T21 group than in euploid pregnancies (*p* < 0.001). We did not notice any significant difference in plasma A1AT between the study and control groups (Figure 1).

Table 1 substantiates all the parameters analyzed with the concentrations found in T21 and control samples and the statistical comparison results (Table 1). No differences were observed between T21 plasma and amniotic fluid 25-OH vitamin D concentrations (*p* > 0.05). However, 25-OH vitamin D in the control group was higher in amniotic fluid than in plasma samples (*p* < 0.01). In the case of asprosin and DNA/RNA OSDP, no differences were noted between plasma samples and amniotic fluid in either euploid pregnancy or T21 groups (*p* < 0.05). AGE and A1AT concentrations in the control and T21 groups were higher in plasma than in amniotic fluid (AGE: *p* < 0.01; *p* < 0.0001; A1AT: *p* < 0.001; *p* < 0.0001, respectively). Control group IMA concentrations were lower in amniotic fluid than in plasma samples (*p* < 0.0001). 

### 3.2. Correlations between Examined Parameters

**Figure 2:**
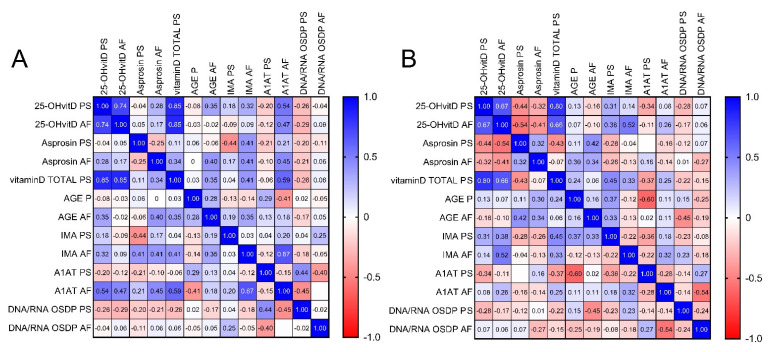
Graphical Spearman correlation matrix of the biochemical parameters in (**A**) the control group and (**B**) the study group. A1AT, alfa-1-antitrypsin; AF, amniotic fluid; AGE, advanced glycation end products; IMA, ischemia-modified albumin; OSDP, oxidative stress damage product; PS, plasma; Vit D, vitamin D.

Spearman coefficients to describe the relationships between the studied parameters were calculated; the obtained results are presented on correlation matrices (Figure 2). Among the biochemical parameters measured in the control group, positive correlations were observed between total plasma vitamin D and plasma 25-OH vitamin D (*r* = 0.85; *p* < 0.001), and between plasma total vitamin D and amniotic fluid A1AT (*r* = 0.59; *p* < 0.05). Additionally, positive correlations were noticed between the control group’s amniotic fluid 25-OH vitamin D and A1AT (*r* = 0.47; *p* < 0.05), as well as between amniotic fluid asprosin and A1AT (*r* = 0.45; *p* < 0.05). Accordingly, a positive correlation was demonstrated between A1AT and DNA/RNA OSDPs measured in plasma (*r* = 0.44; *p* < 0.05). A negative correlation was also demonstrated between amniotic fluid A1AT and plasma DNA OSDPs in the control group (*r* = −0.45; *p* < 0.05) (Figure 2A).

Considering the study group, strong positive correlations were observed between plasma total vitamin D and 25-OH vitamin D (*r* = 0.80; *p* < 0.001), as well as between plasma total vitamin D and plasma IMA (*r* = 0.45; *p* < 0.05). A positive correlation between amniotic fluid IMA and amniotic fluid 25-OH vitamin D was observed in the study group (*r* = 0.52, *p* < 0.05). Negative correlations between amniotic fluid 25-OH vitamin D and plasma asprosin measurements (*r*= −0.54 *p* < 0.05), along with plasma AGE and plasma A1AT, were also demonstrated (*r* = −0.60; *p* < 0.05). Additionally, a negative correlation between the T21 group’s amniotic fluid DNA/RNA OSDPs and amniotic fluid A1AT was observed (*r* = −0.54; *p* < 0.05) (Figure 2B). No significant correlation was observed between the plasma and the amniotic fluid for the corresponding parameters, either in the control or the study group.

### 3.3. Screening Utility of the Tested Parameters

**Figure 3:**
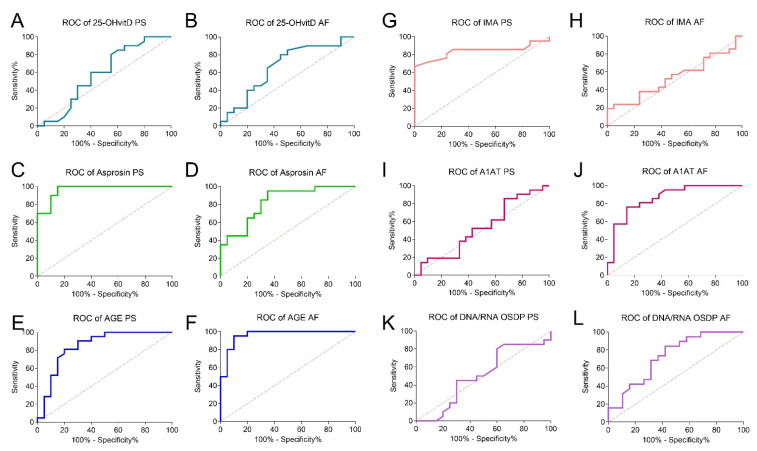
ROC curves of the studied parameters. (**A**) Plasma 25-OH vitamin D; (**B**) amniotic fluid 25-OH vitamin D; (**C**) plasma asprosin; (**D**) amniotic fluid asprosin; (**E**) plasma advanced glycation end products; (**F**) amniotic fluid advanced glycation end products; (**G**) plasma ischemia-modified albumin; (**H**) amniotic fluid ischemia-modified albumin; (**I**) plasma alfa-1-anitrypsin; (**J**) DNA/RNA oxidative stress damage product; (**K**) plasma DNA/RNA oxidative stress damage products; (**L**) amniotic fluid DNA/RNA oxidative stress damage products. AF, amniotic fluid; PS, plasma; OS, oxidative stress.

To determine the diagnostic utility of the tested parameters, the ROC curve was calculated (Table 2), and an illustration of the relationship between sensitivity and specificity is presented in the ROC graphs (Figure 3). The cutoff values were set using Youden’s index. The highest sensitivity was observed for plasma and amniotic fluid asprosin, as well as amniotic fluid AGE (1.00; 0.95; and 0.95, respectively). Plasma IMA and amniotic fluid AGE demonstrated the highest specificity in the T21 screening (1.00 and 0.90, respectively). Differences regarding the chance of detection (OR) for T21 patients based on the studied parameter concentrations are also shown. Relationships between T21 occurrence and amniotic fluid asprosin (OR 22.78), AGE (OR 2.11), IMA (OR 0.18) and plasma asprosin (OR 8.20) and A1AT (OR 5.75) concentrations were noted (*p* < 0.05).

To evaluate the diagnostic usefulness of asprosin, A1AT, IMA, AGE, and DNA/RNA OSDPs as prenatal screening tools, the areas under the ROC curves (AUCs) were calculated and compared to AUC = 0.50 (borderline of the diagnostic usefulness of a test). Asprosin and A1AT demonstrated the highest screening value. The highest AUC value was demonstrated for plasma asprosin (0.97; *p* < 0.0001) and amniotic fluid AGE (0.96; *p* < 0.001). The amniotic fluid A1AT assay was characterized by AUC = 0.87 (*p* < 0.001). Furthermore, the AUC value of the DNA/RNA OSDP concentration in amniotic fluid samples was calculated to be AUC = 0.73 (*p* < 0.05). The 25-OH vitamin D (both in plasma and amniotic fluid), amniotic fluid IMA, plasma A1AT, and DNA/RNA OSDPs concentrations demonstrated no diagnostic usefulness in T21 screening (*p* > 0.05) (Figure 3).

## 4. Discussion

### 4.1. Main Findings

In our study, we determined the T21 screening utility of DNA/RNA OSDPs, as well as other commonly used oxidative stress markers: IMA and AGE. Furthermore, novel antioxidant proteins—asprosin and A1AT—and vitamin D were also assessed and compared between T21 and euploid pregnancies. To the best of our knowledge, this is the first comparative analysis of oxidative stress biomarkers in prenatal T21 in both amniotic fluid and maternal plasma. Significant differences in plasma asprosin, AGE, and IMA, as well as amniotic fluid asprosin, AGE, DNA/RNA OSDPs, and A1AT, were observed between T21 and euploid pregnancies, suggesting the substantial role of oxidative stress in T21 pathology. Referring to the fact that the maternal compartment is constantly connected to the fetus [23,24,25], these parameters were analyzed in maternal plasma to determine the potential screening utility. Moreover, a concentration comparison between maternal plasma and amniotic fluid was evaluated to determine the insufficient metabolic pathway origins during T21 prenatal development. 

It has been noticed that upregulated oxidative stress levels in T21 pathogenesis may result in the oxidation of polyunsaturated fatty acids, and therefore induce cell membrane-destructive effects. This oxidation process has been suggested as one of the major causes of cognitive disabilities observed in this disease [13]. Studies have also indicated that an increased level of oxidative stress results in DNA injury, cytoskeletal and chromatin reorganization, defects in apoptotic cell pathway, and aberrant cell cycle checkpoint function [15,26,27,28,29]. To evaluate the degree of DNA damage and the effectiveness of DNA repair processes in T21 pregnancy, concentrations of the DNA/RNA OSDPs, such as 8-OHG, 8-OHdG, and 8-hydroxyguanine, were determined in both plasma and amniotic fluid samples [30]. Our results showed an increased level of amniotic fluid DNA/RNA OSDPs measured in the study group. The lack of a strong correlation observed between DNA/RNA OSDPs and other oxidative stress markers suggests that oxidative stress in T21 pregnancy is a multifactorial and complex process [31]. Referring to the fact that we did not observe any significant difference in the maternal plasma DNA/RNA OSDPs between the study and control groups, it could be hypothesized that the processes associated with increased oxidative stress are more likely related to disturbed metabolic pathways in the fetal compartment. Interestingly, deregulated measurements were detected mainly in the amniotic fluid and did not transfer through maternal circulation. Additionally, the upregulated oxidative stress levels are a potentially important link of the pathological mechanism of abnormal fetal development [26,32].

Following the increased oxidative stress status in T21 pregnancy, we also evaluated the antioxidant state in which the key modulator is considered to be vitamin D. Despite bone mineralization, vitamin D is also involved in many biological processes, such as immune system modulation and antioxidation [33,34]. Vitamin D components can be divided into five types (D1–D5); their biological functions are triggered by 1.25 OH vitamin D, which is activated in the mitochondria from the 25-OH form [35]. Vitamin D supplementation is associated with a decrease in oxidative stress, improvement in anti-inflammatory defense, and activation of DNA repair processes [36,37]. In 2017, Zubillaga et al. proved that adults with T21 are at greater risk of vitamin D deficiency, and the additional supplementation brings beneficial results [38]. Palacios et al. showed that vitamin D supplementation is necessary for decreasing the risk of pregnancy-related abnormalities, including pre-eclampsia, preterm birth, decreased birth weight, and other related diseases [35,36,38,39,40,41,42]. In our study, decreased levels of vitamin D were found among women carrying T21 fetuses, similar to the results received in T21 individuals [41]. The data obtained in the study showed decreased 25-OH vitamin D concentrations below the recommended level (<30 ng/mL) [32]. Furthermore, decreased vitamin D concentrations suggest insufficient antioxidant potential in the maternal compartment, which may result in a more severe subsequent course of fetal comorbidities [43]. Accordingly, in our study, the decreased 25-OH vitamin D concentration observed in T21 pregnancies was found to be positively correlated with another antioxidant and anti-inflammatory protein—A1AT [44]. A1AT, also known as serpin 1, protects neurons and glial cells from oxidative stress and glucose deprivation [45,46]. It is known that A1AT deficiency is a rare disease that significantly increases the risk of serious lung and/or liver diseases [47]. In our research, the concentration of amniotic fluid A1AT was significantly lower in the study group compared to the control group. The results suggest that a decrease in the A1AT concentration combined with aggravated inflammation processes and oxidative stress observed in T21 pregnancy may negatively impact plural comorbidities and the occurrence of fetal malformations [48,49,50]. A1AT deficiency combined with the decreased vitamin D levels observed in our study could have a multitude of effects of deregulated paths in T21 pregnancy development [44]. Furthermore, the negative correlation demonstrated between DNA/RNA OSDPs and A1AT showed that an increased degree of oxidative stress is combined with A1AT deficiency observed in amniotic fluid. Nevertheless, we did not observe any significant difference in the A1AT plasma concentration between T21 and euploid pregnancies. 

In our study, elevated levels of the novel antioxidant protein asprosin were found in T21 amniotic fluid and plasma compared to those in the euploid control group. Asprosin is a hormone secreted by white adipose tissue activated by fasting as a response to low plasma glucose concentrations [51]. Interestingly, Zhang et al. proved that asprosin upregulates the activity of the antioxidant enzyme superoxide dismutase 2, which is associated with a decrease in the concentration of reactive oxygen species (ROS) and apoptosis processes [52]. An increased asprosin concentration may result from antioxidant maternal protection related to the developing T21 fetus [51,52,53,54]. Vitamin D deficiency is inversely associated with asprosin concentrations, which, combined with increased DNA/RNA OSDP levels, confirms that the antioxidant deficiency caused by the developing T21 fetus is insufficiently counteracted by the maternal organism.

The results of the present study are convergent with those obtained by other authors. Perrone et al. suggested that an increased oxidative stress level is detectable in amniotic fluid samples in early T21 pregnancy. In their study, upregulated isoprostane concentrations, a novel marker of free radical-catalyzed lipid peroxidation related to increased oxidative stress, were noticed. Their hypothesis, based on these outcomes, referred to the fact that T21 fetal development is interrupted by an environment with increased oxidative stress, which may injure many tissues [32]. These results were updated by Perlugi et al., where decreased levels of glutathione (GSH) were observed and significantly increased levels of several markers of oxidative stress were found in T21 amniotic fluid. The sources of oxidative stress in pregnancy can be various, from the placenta to maternal and fetal tissues, and the induction of oxidative stress reactions could also come from external factors [55].

No significant correlations between corresponding parameters in plasma and amniotic fluid were demonstrated. We found it confusing that studied parameter concentrations in the amniotic fluid were not directly related (proportional) to the concentrations in the maternal plasma—in a number of cases, higher maternal content was not readily translated into higher concentrations in the amniotic fluid. This would also indicate that the relationship between maternal and fetal oxidative stress is complex beyond a simple diffusion. The source of oxidative stress in pregnancy manifests in the placenta, in particular, but also originates from maternal and/or fetal cells and external factors [55]. It can be hypothesized that the correlation is described by some monotonic, but not linear function. Studies on the transfer mechanisms between the maternal and fetal compartment are needed to determine the association between the parameters studied in the amniotic fluid and maternal plasma.

### 4.2. Strength and Limitations

It was previously hypothesized that the upregulated oxidative stress level is related to T21 pathogenesis; this was later proved by Žitňanová et al., who measured and reported upregulated levels of oxidative stress markers in T21 individuals [22]. Thus, it seemed necessary to evaluate the hypothesis about oxidative stress biomarkers in T21 prenatal screening. The results indicate a potential role of the application of oxidative stress markers in the pre-natal screening of T21 with the highest screening utility of plasma asprosin. Moreover, the origins of the disturbed metabolic pathways were analyzed. Our study indicates that oxidative stress-related parameters in the maternal plasma were not directly related to concentrations in the amniotic fluid. It seems that disturbed metabolic processes in the fetal compartment are not particularly counteracted by additional syntheses of antioxidant substances in maternal circulation [38]. Furthermore, the analyzed protein’s direct functions as antioxidants were not thoroughly examined. In this case, our study has indicated the novel possibilities in basic research, especially referring to the fact that insufficient antioxidants properties were established during T21 fetus development. These analyses are of great importance in understanding the role of oxidative stress in the pathophysiology of T21. Furthermore, the number of studies performed on T21 individuals to establish the negative impact of increased oxidative stress status is still insufficient. Preclinical studies concerning the impact of oxidative/antioxidative state on the development of T21 are still needed [56]. However, in our study, the low diagnostic utility of measurements of the oxidative stress marker IMA in T21 pregnancy were demonstrated. Although IMA and AGE have never been measured in T21 pregnancy before, extensive data in the literature suggest their promising diagnostic utility in pre-eclampsia and pregnancy hypertension [57,58]. Despite confirmation of a higher level of oxidative stress, the IMA and AGE levels have been shown to not be remarkably increased in various complications related to T21 gestation [34,42,59,60,61]. Moreover, referring to the limited size of the experimental group, further evaluation and data validation using a larger cohort are required to confirm the diagnostic usefulness of the studied oxidative stress parameters.

### 4.3. Implications and Future Perspectives

Considering that oxidative stress markers are still investigated for their possible screening utility, the oxidative stress markers in T21 pregnancy screening were evaluated. The commonly used noninvasive prenatal test for calculating the risk of T21, which combines ultrasound markers with biochemical markers of pregnancy-associated plasma protein A (PAPP-A) and serum-free human chorionic gonadotropin (B-HCG), is characterized by 93% accuracy. The separate diagnostic utility in maternal plasma has been proven (AUC for PAPP-A = 0.777; AUC for B-HCG = 0.668; AUC for combined PAPP-A + B-HCG = 0.8533) [62]. Comparing these data to the plasma asprosin measurement, characterized by AUC = 0.965, the diagnostic utility of maternal plasma asprosin as a potential noninvasive marker in T21 prenatal screening was demonstrated. Moreover, these results are also comparable with the free fetal DNA measurement, characterized by 99% accuracy [9]. Additionally, the possible association of the occurrence of T21 comorbidities and prenatal determination of asprosin in follow-up studies should be evaluated. The OR calculation has shown that deregulated concentration of plasma and amniotic fluid asprosin, A1AT, and amniotic fluid IMA during the second trimester increased the risk of Down syndrome among pregnant women (*p* < 0.05).

The preventive effects of antioxidants counteracting the harmful impact of ROS or acting as treatment for oxidative stress-related diseases are still constantly being examined. Accordingly, the potential beneficial effect of antioxidant administration during T21 development could reduce the cognitive and neuronal dysfunctions associated with T21 [63]. The evidence from studies performed in vitro and in vivo to evaluate the positive effects of dietary antioxidants seems compelling [56,64,65]. However, nonconclusive results in this area clearly demonstrate that more attention should be paid to the performance of high-quality randomized controlled trials [64,65,66,67]. Despite this, Nachvak et al. proved that alpha-tocopherol supplementation decreases the levels of oxidative stress markers in T21 [68]. Furthermore, antioxidant supplementation in adult T21 individuals could slow the development of dementia and Alzheimer’s disease, which are the most strongly related to T21 diseases. Our results confirm the antioxidant deficiencies of pregnant women with fetal T21 and the potential of antioxidant treatment of pregnant women. In this case, this study has uncovered novel targets for evaluations in future preclinical trials [56,69]. Despite the relevant value of our research, this study should be considered as preliminary. In future research, long-term follow-up studies performed on large cohort study groups are of utmost importance.

## 5. Conclusions

The diagnostic utility in the prenatal screening of T21 of plasma measurements of asprosin, IMA, AGE, and DNA/RNA OSDPs was demonstrated. The obtained results indicate a potential role of the application of oxidative stress markers in the prenatal screening of T21, with the highest screening utility of asprosin measurement. Decreased A1AT with vitamin D and increased asprosin and DNA/RNA OSDP concentrations are related to T21 development. However, based on the present study, it is reasonable to speculate that oxidative stress occurs in the T21 fetal compartment rather than in the maternal compartment, and the maternal organism is inefficient in overcoming the antioxidant deficiencies caused by the developing T21 fetus. Thus, antioxidant applications in T21 pregnancy should still be evaluated.

## Figures and Tables

**Table 1:** Basic statistics and comparison of studied protein concentrations measured in the plasma and amniotic fluid samples.

Marker	Material	Study Group	Unit	Median Value	Min	Max	*p* Value (Control vs. T21)	*p* Value (between Study Material)	
								Control PS vs. Control AF	T21 PS vs. T21 AF
**25-OH vitamin D**	PS	Control	mg/mL	22.22	14.00	35.92	NS	*p* < 0.01	NS
		T21		19.51	14.24	30.44			
	AF	Control		30.60	14.24	51.34	NS		
		T21		25.20	12.59	42.07			
**Asprosin**	PS	Control	ng/mL	10.57	4.45	15.17	*p* < 0.0001	NS	NS
		T21		17.28	12.94	26.59			
	AF	Control		10.87	4.01	17.03	*p* < 0.0001		
		T21		15.53	8.09	24.77			
**AGE**	PS	Control	ng/mL	12.96	4.96	26.03	*p* < 0.001	*p* < 0.01	*p* < 0.0001
		T21		9.16	4.52	13.01			
	AF	Control		8.27	3.06	11.55	*p* < 0.0001		
		T21		3.00	1.67	4.89			
**IMA**	PS	Control	µg/mL	6.79	5.00	12.00	*p* < 0.0001	*p* < 0.0001	NS
		T21		3.61	0.90	22.52			
	AF	Control		2.64	1.05	9.34	NS		
		T21		2.28	0.33	6.23			
**A1AT**	PS	Control	mg/L	1.98	0.95	3.38	NS	*p* < 0.001	*p* < 0.0001
		T21		1.95	1.26	1.69			
	AF	Control		0.49	0.08	2.90	*p* < 0.0001		
		T21		0.18	0.01	0.56			
**DNA/RNA OSDP**	PS	Control	pg/mL	37.81	23.21	46.83	NS	NS	NS
		T21		37.57	28.53	58.40			
	AF	Control		31.16	12.64	45.22	*p* < 0.05		
		T21		38.48	27.06	51.46			

A1AT, alfa-1-antitrypsin; AF, amniotic fluid; AGE, advanced glycation end products; IMA, ischemia-modified albumin; NS, not significant; OSDP, oxidative stress damage product; PS, plasma; T21, trisomy 21.

**Table 2:** Diagnostic criteria of the receiver operating characteristic (ROC) curve for the tested parameters.

Marker	Unit	AUC	*p* (AUC = 0.50)	Cut Off Value	Sensitivity	Specificity	OR	*p*
**25-OH vitamin D PS**	mg/mL	0.59	NS	<26.18	0.85	0.40	1.62	NS
**25-OH vitamin D AF**	mg/mL	0.66	NS	<31.21	0.85	0.50	3.27	NS
**Asprosin PS**	ng/mL	0.97	<0.0001	>12.70	1.00	0.85	8.20	*p* < 0.05
**Asprosin AF**	ng/mL	0.83	<0.001	>12.91	0.95	0.65	22.78	*p* < 0.05
**AGE PS**	ng/mL	0.85	<0.001	<11.00	0.81	0.80	1.00	NS
**AGE AF**	ng/mL	0.96	<0.0001	<4.184	0.95	0.90	2.11	*p* < 0.05
**IMA PS**	µg/mL	0.84	<0.001	<4.798	0.67	1.00	1.05	NS
**IMA AF**	µg/mL	0.54	NS	<1.798	0.38	0.76	0.18	*p* < 0.05
**A1AT PS**	mg/L	0.53	NS	<2.341	0.81	0.33	5.75	*p* < 0.05
**A1AT AF**	mg/L	0.87	<0.0001	<0.3180	0.76	0.86	0.71	NS
**DNA/RNA OSDP PS**	pg/mL	0.51	NS	<40.30	0.80	0.40	3.27	NS
**DNA/RNA OSDP AF**	pg/mL	0.73	<0.05	>31.76	0.84	0.58	3.78	NS

A1AT, alfa-1-antitrypsin; AF, amniotic fluid; AGE, advanced glycation end products; AUC, area under the received operative characteristic (ROC) curve; IMA, ischemia-modified albumin; NS, not significant; OR, odds ratio; OSDP, oxidative stress damage product; PS, plasma.

## Data Availability

Not applicable.
